# High Flow Conditions Increase Connexin43 Expression in a Rat Arteriovenous and Angioinductive Loop Model

**DOI:** 10.1371/journal.pone.0078782

**Published:** 2013-11-13

**Authors:** Volker J. Schmidt, Johannes G. Hilgert, Jennifer M. Covi, Christian Weis, Johanna O. Wietbrock, Cor de Wit, Raymund E. Horch, Ulrich Kneser

**Affiliations:** 1 Department of Plastic and Hand Surgery, University Hospital Erlangen, Friedrich-Alexander-Universität Erlangen-Nürnberg, Erlangen, Germany; 2 Department of Hand-, Plastic- and Reconstructive Surgery, BG Unfallklinik Ludwigshafen, Universität Heidelberg, Heidelberg, Germany; 3 Department of Physics, Friedrich-Alexander-Universität Erlangen-Nürnberg, Erlangen, Germany; 4 Department of Physiology, Universität zu Lübeck, Lübeck, Germany; King’s College London School of Medicine, United Kingdom

## Abstract

Gap junctions are involved in vascular growth and their expression pattern is modulated in response to hemodynamic conditions. They are clusters of intercellular channels formed by connexins (Cx) of which four subtypes are expressed in the cardiovascular system, namely Cx37, Cx40, Cx43 and Cx45. We hypothesize that high flow conditions affect vascular expression of Cx in vivo. To test this hypothesis, flow hemodynamics and subsequent changes in vascular expression of Cx were studied in an angioinductive rat arteriovenous (AV) loop model. Fifteen days after interposition of a femoral vein graft between femoral artery and vein encased in a fibrin-filled chamber strong neovascularization was evident that emerged predominantly from the graft. Blood flow through the grafted vessel was enhanced ∼4.5-fold accompanied by increased pulsatility exceeding arterial levels. Whereas Cx43 protein expression in the femoral vein is negligible at physiologic flow conditions as judged by immunostaining its expression was enhanced in the endothelium of the venous graft exposed to these hemodynamic changes for 5 days. This was most likely due to enhanced transcription since Cx43 mRNA increased likewise, whereas Cx37 mRNA expression remained unaffected and Cx40 mRNA was reduced. Although enhanced Cx43 expression in regions of high flow in vivo has already been demonstrated, the arteriovenous graft used in the present study provides a reliable model to verify an association between Cx43 expression and high flow conditions in vivo that was selective for this Cx. We conclude that enhancement of blood flow and its oscillation possibly associated with the transition from laminar to more turbulent flow induces Cx43 expression in a vein serving as an AV loop. It is tempting to speculate that this upregulation is involved in the vessel formation occuring in this model as Cx43 was suggested to be involved in angiogenesis.

## Introduction

Blood vessels enable oxygen supply and delivery of nutrients for multicellular organisms. To fulfil these tasks and meet required tissue demands the vascular system is able to adapt by distinct mechanisms that act on different time scales. Acutely, vessels constrict or dilate thereby adjusting vascular resistance and thus regulating local blood flow to tissues in a wide dynamic range. Herein, mechanical forces exerted by the flowing blood onto the vessel wall in conjunction with signals derived from the tissue are key players. Importantly, vasomotor signals are transmitted along the vessel length through intercellular channels which are provided by vascular gap junctions, thus coordinating the cellular behaviour in the microcirculation [1]–[5]. On a longer-time scale, the vasculature grows or shrinks, reshapes the network and, notably, creates new vessels from preexisting vessels, thereby allowing tissue growth and regeneration. Mechanical forces as well as signals derived from the tissue, such as pro- and antiangiogenic molecules, are underlying mechanisms to stimulate the adaptation of the network and vessel structure [6], [7].

Recent findings suggest that gap junctional communication is also critically involved in vascular growth and angiogenesis [8]–[11]. Gap junctions are composed of proteins termed connexins, of which six oligomerize to form a channel within the plasma membrane that docks to its counterpart in an adjacent cell creating an intercellular pore that is sealed against the external environment facilitating the propagation of electrical and chemical signals between the cells of the vessel wall [12]. The connexin protein family comprises 20 members that are classified according to their theoretical molecular mass. In the cardiovascular system, four Cx isoforms (Cx37, Cx40, Cx43, and Cx45) are expressed [13], [14]. Of these, Cx43 is of special importance in uterine angiogenesis during pregnancy, as its specific deletion impairs development of new blood vessels in mice, resulting in the arrest of embryo growth [8]. Furthermore, down-regulation of Cx43 by means of Cx43-specific small interference RNA decreases proliferation and angiogenesis in human aortic endothelial cells in vitro [9], [11]. Different studies aimed to investigate a potential correlation of vascular expression of Cx and varying flow conditions. In vitro approaches revealed an induction of Cx40 upon enhanced shear stress and blood flow [15]. In vivo, the expression of Cx40, which is the most important endothelial Cx at the functional level [16], is likewise regulated by flow. Cx40 was suggested to be central for arterial identity of blood vessels and exerts important functions during arteriogenesis [17]. On the other hand, vascular Cx43 gene expression, which is of special interest in terms of angiogenesis, was enhanced in vitro by mechanical loads, either shear stress or mechanical stretch [18]. In the intact animal Cx43 is extremely abundant in endothelial cells localized at the edge of ostia of branching vessels and at flow dividers representing areas of disturbed blood flow and shear stress [17]. Moreover, Cx43 is expressed in thoracic aortic endothelium whereas it is undetecable in endothelia of other vessels [19] in which blood flow is presumably less oscillatory and more laminar. Aim of the present study is to investigate whether there is a direct correlation between expression of connexins and high and/or altered flow conditions in vivo by means of an arteriovenous graft that provides a reliable model for modulating blood flow conditions in vivo.

Previously, we have established a unique model of angiogenesis *in vivo* which is distantly related to the early work of Erol and Spira [20]. It is based on hemodynamic forces exerted on a grafted vessel that is interpositioned between the femoral artery and vein creating an arteriovenous (AV) loop. In such grafted vessels embedded in a fibrin matrix, angiogenesis occurs without the need of exogenous addition of angiogenic factors [21], [22]. Importantly and in contrast to other models of angio- or vasculogenesis, angiogenic factors are also not released endogenously from surrounding tissues that are commonly made ischemic during the experiment and thus initiate hypoxia-dependent angiogenic signalling. The independency of angiogenic factors renders the AV loop an unique model of angiogenesis in vivo and suggests that mechanical forces are crucial in this model which may act through modulation of Cx expression.

We hypothesize, that changes in mechanical forces exerted by alterations in blood flow pattern modulate the expression of connexins within the vascular wall in vivo and may change the vascular identity of a vessel (artery vs. vein). To test this hypothesis, blood flow was characterized in the rat AV loop model and vascular expression of Cx was assessed at the mRNA and protein level in a vein used as interpositioned vessel.

## Materials and Methods

### Experimental Design

Experiments were performed in accordance with the German Animal Welfare Act and approved by the Institutional Animal Care and Use Committee of the Regierungspräsidium Mittelfranken (54–2532.1–34/09). 39 male Lewis rats (Charles River Laboratories) at the age of 2–4 month weighting 280–385 g were anaesthetized using isoflurane (Baxter). An arteriovenous (AV) loop was constructed between the left femoral artery and vein by interposition of a venous graft from the contralateral thigh. The AVL was either placed subcutaneously or embedded within a fibringel-filled chamber to induce three-dimensional angiogenesis. Angiogenesis was investigated 15 days after surgery at the previously estimated time of new vessel formation [21]–[23].

### Isolation Chamber and Fibrin Matrix

The used cylindrical chamber exhibited an inner diameter of 10 mm and a height of 6 mm (P. Greil, Department of Materials Science, Glass and Ceramics, University of Erlangen-Nuremberg). Four centrally placed tubes prevented displacement of the loop. The chamber was filled with 1000 µL fibrin sealant (Tissucol®; Baxter Health Care S.A.) containing fibrinogen (10 mg/mL), thrombin (2 IU/mL) and aprotinin (1500 KIE/mL) to prolong fibrinolysis [23]. Detailed chamber design and surgical placing of the AV loop are described elsewhere [24].

### Surgical Procedures

All operations were performed using a surgical microscope (magnification × 20, OPMI IFC, Carl Zeiss) and by the same investigator (J.C.). After induction of anaesthesia (5% Isoflurane; Baxter), animals received tramadol (7.5 mg/kg i.v.; Tramal®; Grünenthal), benzylpenicillin-streptomycin (0,5 mL/kg i.m.; Veracin®-compositum, Albrecht) and heparine (80 IU/kg Liquemin®; Ratiopharm). After midventral incision of the medial left thigh, femoral artery and vein were dissected and separated from the pelvic artery downstream to the bifurcation of the femoral artery at knee level. A 20 mm femoral vein graft was harvested from the contralateral thigh and interposed between the left femoral artery and vein by microsurgical techniques using 11/0 sutures (Ethilon, Ethicon). Thereafter, the entire AV loop was either placed subcutaneously or embedded into the isolation chamber by placing the AV loop carefully around the four plastic tubes. The chamber was filled (see above), closed and fixed onto the underlying adductor fascia (Prolene 3/0, Ethicon). Finally, the skin was closed using Vicryl 3/0 and 4/0 (Ethicon).

The animals were housed in the animal facility of the University of Erlangen Medical Centre and kept at a 12 h dark/light cycle with free access to standard chow (Sniff) and water. At the end of the experiment animals were sacrified by intracardial injection of a combination of embutramid, mebezonium and tetracain (15 ml/kg: T61®: Intervet) under deep general anaesthesia (5% isoflurane).

### Histological and Micro-CT Analysis

For conventional vessel visualization the distal descending aorta was cannulated (24-gauge catheter) after abdominal midline incision. The vasculature was flushed using 100 mL isotonic salt solution containing heparin (100 IE/mL) before 30 mL warmed (37°C) India ink solution [50% v/v India ink (Rohrer&Klinger) in 5% gelatin and 4% mannitol] were injected into the aorta to visualize perfused vessels around the constructed AV loop. For micro-CT analyses, 20 mL yellow Microfil (MV-122) containing 5% of MV Curing Agent (both from Flowtech) was applied instead.

After explantation, constructs were fixed using formaldehyde (3,5%), dehydrated and embedded in paraffin. Histological slices with a thickness of 3 µm were obtained at two standardized planes (500 µm proximal and distal of the central plane) perpendicular to the longitudinal AV loop axis using a microtome (Leica Microsystems). Slices were stained by hematoxylin and eosin according to standard protocols and visualized using conventional microscopy (Olympus IX81, 10× magnification). Subsequent processing of the images was performed using Olympus cellSens dimension (Olympus).

Micro-CT scans were acquired on a high resolution, cone-beam micro-CT scanner developed at the Institute of Medical Physics, University Erlangen, Germany (ForBild). The image-enhancing algorithms and technical specifications are described in detail elsewhere [21].

### Immunohistochemistry

For whole-mount immunostaining of Cx the femoral artery and vein were carefully removed after sacrificing the animal 5 days post-surgery. All vessels were opened longitudinally and pinned flat, luminal-side up. After fixation (5% formaldehyde, 5 min) and washing (PBS), specimens were blocked and permeabilized (1% BSA, 0.2% TritonX-100 in PBS, 2 hours). The preparation was then incubated with the primary antibody (anti- Cx37 1:400 [CX37A11-A, ACRIS Antibodies], anti-Cx40 1:400 [AB1726, Milipore] or anti-Cx43 1:800 [Invitrogen], in part together with anti-CD31 1:800 [PECAM, Invitrogen]) in blocking solution overnight at 4°C. After washing (1% Triton X-100 in PBS, 1 hour; PBS, 30 min), immunocomplexes were visualized by goat anti-rabbit or donkey anti-mouse IgG (1∶800; Alexa Fluro 488 and 594, Invitrogen). After final washing with PBS (3×30 min) the vessels were mounted flat on a slide using Mowiol (Calbiochem). Staining was visualized by means of confocal laser scanning microscopy (SP5X; Leica Microsystems).

### Measurement of Femoral and AV Loop Blood Flow

By means of a microcirculatory flow probe (transit time flowmeter, TS420, Transonic) blood flow was monitored continuously at the femoral vessels (artery and vein) and at the AV loop after surgery before the transfer into the fibrin filled chamber for at least 10 min. This procedure was repeated 15 days after implantation to assess blood flow in the AV loop before and after passing the chamber. Additionally, blood flow was recorded in femoral vessels not subjected to surgical interposition during topical superfusion of papaverin (1 mM; Paveron N, Linden Arzneimittel Vertrieb) to achieve maximal vasodilation and acquire physiological flow values in these vessels. Data were collected at 240 Hz and stored using commercial data acquisition software (Windaq, Dataq).

### Quantitative PCR (qPCR)

Subcutaneously implanted venous AV loop grafts were harvested 15 days after surgery during isoflurane anesthesia, instantaneously frozen in liquid nitrogen and stored at −70°C until later analysis. A part of the femoral vein, which was dissected and reintegrated into the venous limb of the circulation by microsurgical techniques served as control [sham operated group]. This vessel was treated otherwise similar to those vessels interpositioned between artery and vein.

Frozen tissue was disrupted using a mortar and pestle, total RNA extracted by TRIzol (Invitrogen), and purified with RNeasy kits (Qiagen, Hilden, Germany). After reverse transcription (Omniscript RT Kit, Qiagen), quantitaive PCR (qPCR) was performed using iQ SYBR Green Supermix (BioRad). Connexins and GAPDH as reference gene were detected using the following primers:

Cx37: 5′-TGACAGCAGGTGGGGTGCTCTT-3′ (forward) and 5′-AGCCAGCAGCCTACATCAGTGC-3′ (reverse),

Cx40: 5′- GGGCTACTCCTCAAATCCCC-3′ (forward) and 5′- TTGCCAAGGTTCCCATTCCT-5′ (reverse),

Cx43: 5′-CGTGCCGCAATTACAACAAGCA-3′ (forward) and 5′-TGGAGTTCATGTCCAGCAGCAA-3′ (reverse),

GAPDH: 5′-ACCACCCAGCCCAGCAAGGATA-3′ (forward) and 5′-GCCCCTCCTGTTGTTATGGGGTCT-3′ (reverse).

The comparative CT method was used for quantification of gene expression.

### Statistical Analysis

Data are presented as means ±SEM and compared using unpaired *t* test. For more than 2 groups analysis of variance (one-way ANOVA) followed by the Bonferroni post hoc test was used. Differences were considered significant at a corrected error probability of *P*<0.05.

## Results

### Blood Flow in the Arteriovenous Loop

**Figure 1:**
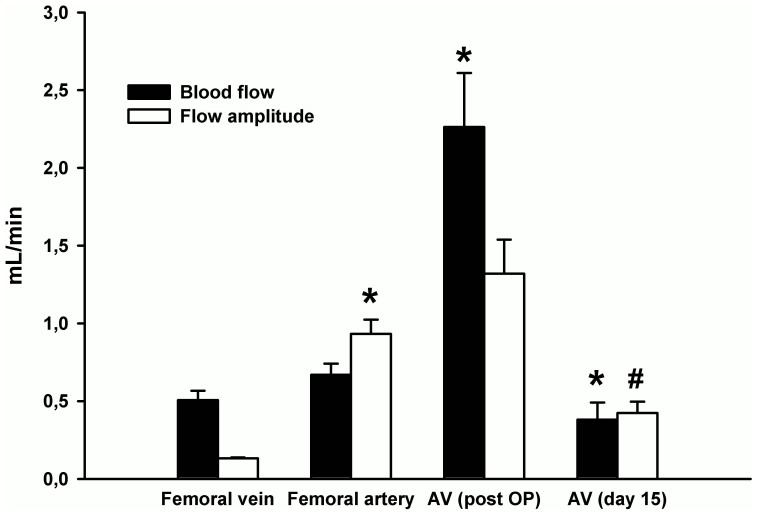
Mean blood flow (black) and mean flow amplitude (white) measured using flow probes are depicted. The venous graft (AV post OP, n = 19) was exposed to a higher median blood flow and enhanced flow amplitude (cardiac-cycle dependent flow difference) compared to its physiologic flow pattern (femoral vein, n = 26) and also compared to the femoral artery (n = 26). After onset of angiogenesis (AV, day 15, n = 9), the mean blood flow and flow amplitude through the AV loop was significantly decreased and ranged slightly below the regular femoral artery flow pattern. *n* indicates the number of animals per group. ^#^ *P*<0.01, *P<0.001 vs. previous group.

High resolution blood flow data was recorded by means of microvascular flow probes in 26 rats. In femoral vessels without arteriovenous (AV) loop, mean blood flow amounted to about 0.67 mL/min in artery and 0.51 mL/min in vein. However, flow was substantially more pulsatile in arteries than in veins which is reflected by the difference between maximal and minimal flow during a cardiac cycle. This flow amplitude was approximately 7 fold larger in the artery (Figure 1). The interposition of a venous graft between artery and vein introduced substantial higher flow through the grafted vessels (∼4.5-fold) and also exposed the venous graft towards a drastically increased pulsatile flow that even exceeded arterial pulsatility (Figure 1).

### Angiogenesis in the Arteriovenous Loop and Subsequent Changes in Hemodynamics

**Figure 2:**
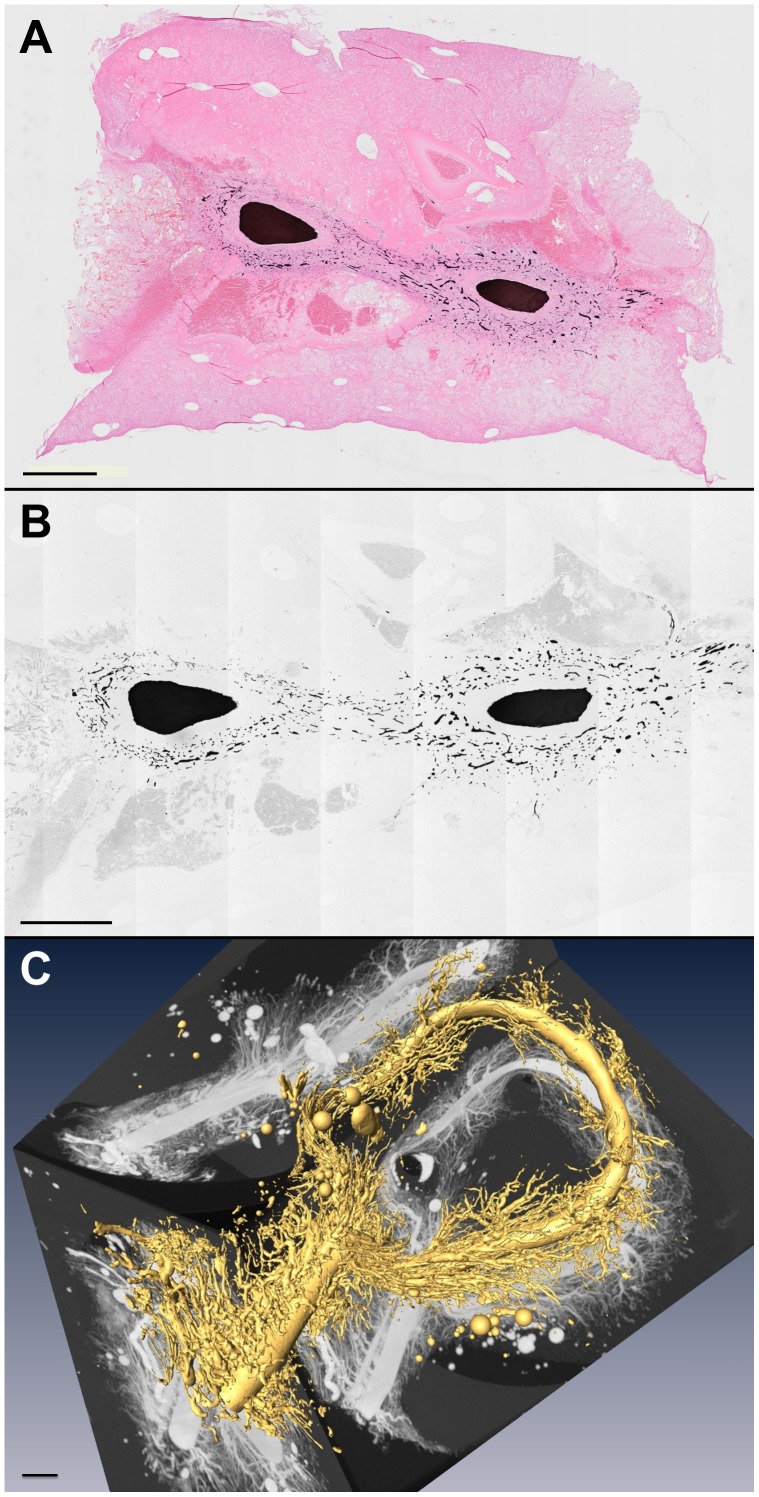
De novo angiogenesis is visualized 15 days following AV loop preparation. After injection of black-appearing India-ink into the aorta cross sections of the explanted and paraffin embedded AV loop construct were obtained perpendicular to the longitudinal AV loop axis which are the central dark areas. Preparations are stained with hematoxylin and eosin (A) or without staining (B). The central AV loop vein graft is surrounded by newly formed and dye filled vessels. Micro-CT analyses following microfil© perfusion (C) confirm the strong luminal neovascularisation arising from the venous graft without the need of additional growth factors. Scale bars: 1000 µm.

**Figure 3:**
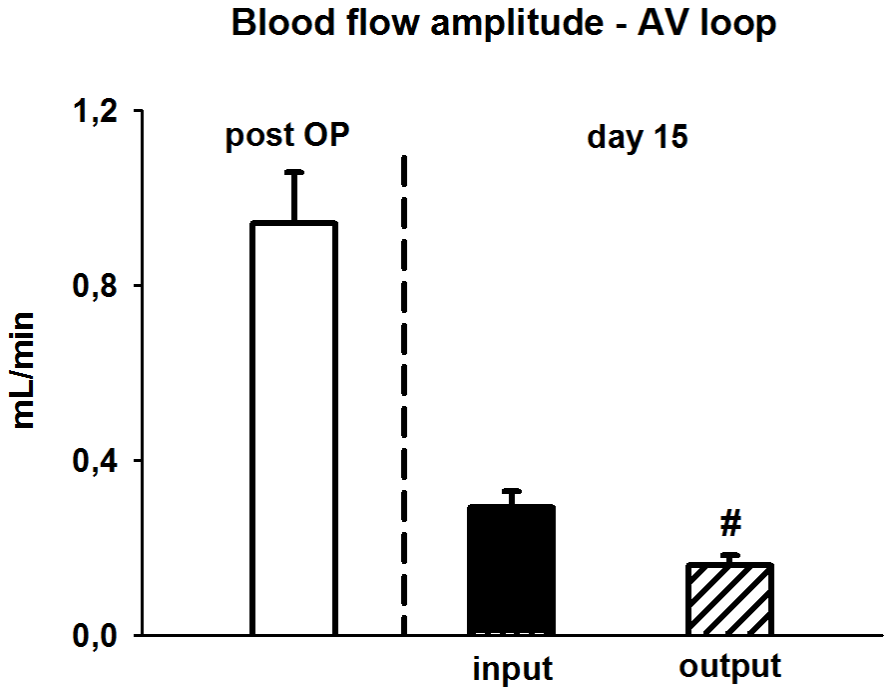
Cardiac-cycle dependent flow differences are depicted as blood flow amplitude. The high blood flow amplitude measured immediately after AV loop implantation (n = 19) decreased after onset of de novo angiogenesis within the AV loop chamber and attained at day 15 levels lower than observed in femoral arteries in situ. Interestingly the flow amplitude was significantly attenuated after passing the AV loop (^#^ *P*<0.05, inflow vs. outflow, day 15, n = 9).

Angiogenesis was monitored by placing the AV loop into an isolated subcutaneous chamber filled with a fibrin matrix. After 15 days without addition of angiogenic compounds, a strong neovascularization was evident within the 3-dimensional construct. The newly formed vessels were perfused as they could be visualized after ink injection (Figure 2A, 2B) and arose from the venous graft (Figure 2C). During this time of angiogenesis, flow through the graft and its enhanced flow amplitude decreased and attained at day 15 levels that were observed in non-treated vessels (Figure 1). Along the loop, the flow amplitude decreased and thus was significantly lower at its exit than at the entry (Figure 3) suggesting that at least part of the flow perfused a newly formed microcirculatory bed.

### Vascular Connexin Expression

**Figure 4:**
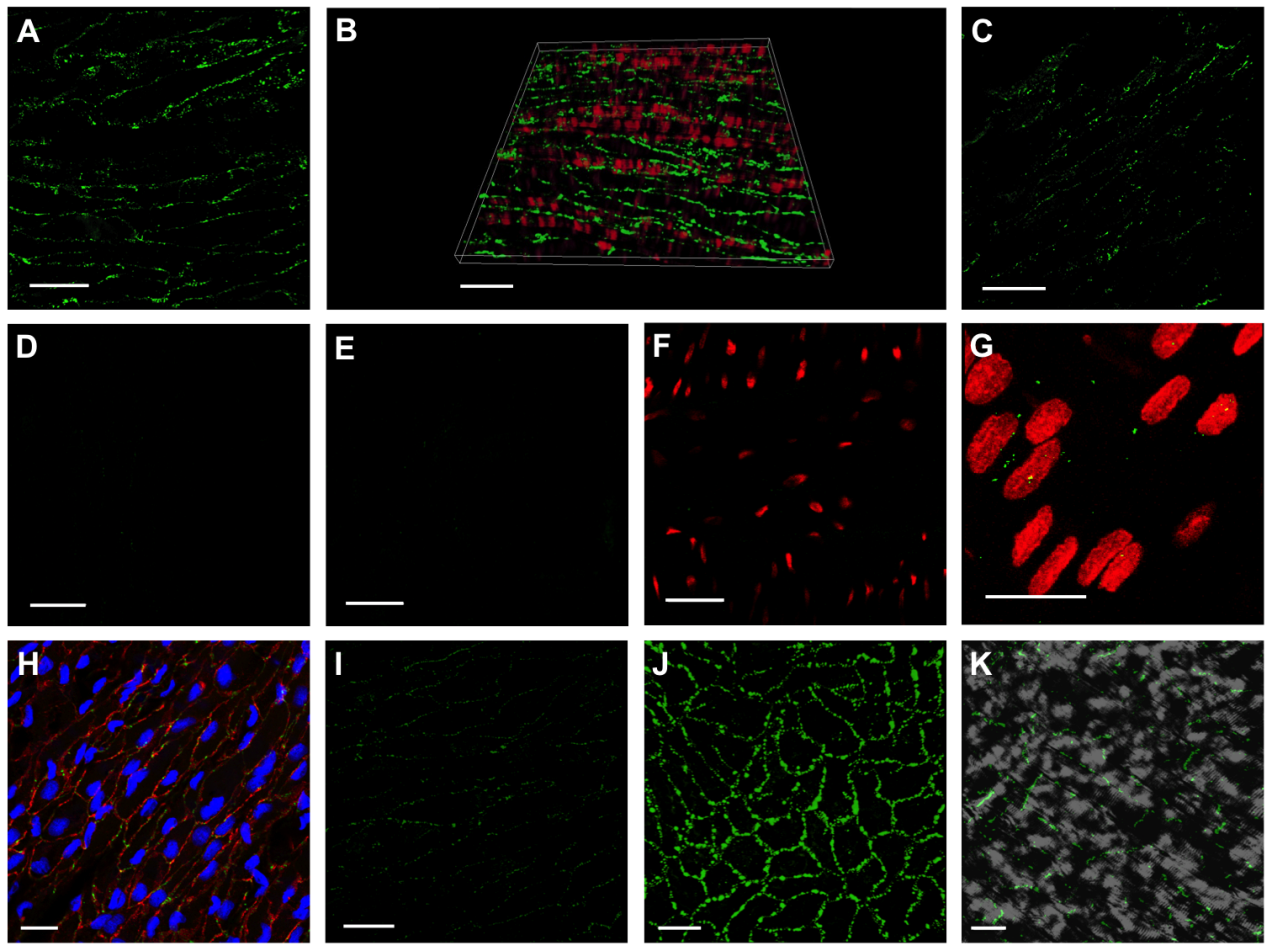
Expression of Cx (green) is visualized in different vessels using confocal laser scanning microscopy. Cx37 (A, B) and Cx40 (C) were located at endothelial cell borders of the femoral artery but staining was weak in the femoral vein (D: Cx37; E: Cx40). Cx43 staining was weak in the femoral artery (F) as well as in the femoral vein (G, red: nuclei staining). In the aorta immunostaining for Cx37 (H), Cx40 (I), and also for Cx43 (J) revealed a pronounced punctate staining at cell borders of endothelial cells. Double staining of Cx37 (H: green) and CD31 (H: red) identifies membranes of endothelial cells by the endothelial marker CD31 which is colocalized with Cx37. Cx43 expression along the junction zones of ventricular myocytes verified the specificity of the Cx43 antibody (K). Scale bars: 20 µm, images obtained using an objective with 63 fold magnification (HCX PL APO CS 63.0×1.30 GLYC, Leica Microsystems GmbH, Germany).

**Figure 5:**
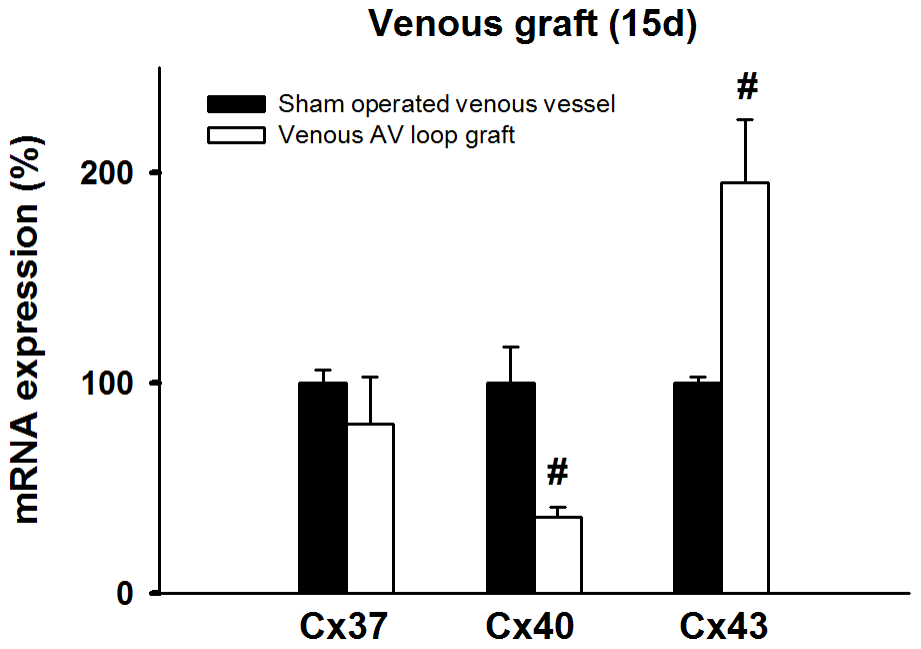
Cxs mRNA expression in venous AV loop grafts after 15 days of hemodynamic stimulation compared to veins that where dissected and reintegrated into the venous limb circulation. RNA expression was normalized to GAPDH and is presented relative to the sham operated group. Compared to the sham operated venous graft which was exposed to physiologic venous flow mRNA expression of Cx43 was substantially enhanced in AV grafted veins. In contrast, the amount of Cx40 mRNA was decreased in AV grafted veins, whereas mRNA expression of Cx37 remained unchanged. ^#^ *P*<0.05 vs. control. n ≥3 animals per group.

The expression of connexins (Cx) was studied in the femoral artery and vein. Vascular cells can be differentiated by simultaneous staining of the nuclei, which are broader in endothelial cells and oriented longitudinally to the vessel axis whereas nuclei in smooth muscle cells that are longitudinally wrapped around the vessel lumen are thinner and oriented perpendicularly [14]. In the femoral artery, Cx37 and Cx40 are expressed in endothelial cells as previously described in mice vessels [25]. The staining pattern was circularly around the centered nuclei suggesting that Cxs are located in the entire plasma membrane of endothelial cells (Figure 4A, 4B and 4C) which was verified by double staining using the endothelial marker CD31 and its colocalization with Cx37 (Figure 4H). In contrast, both, Cx37 and Cx40 were hardly detectable in the vascular wall of femoral veins (Figure 4D and 4E). Expression of Cx43 was weak in both vessels and mostly absent in arteries (Figure 4F) but detectable in the endothelial cell layer of veins (Figure 4G). In contrast to the femoral artery, Cx43 was, in addition to Cx37 (Figure 4H) and Cx40 (Figure 4I) expressed in endothelial cells in the thoracic rat aorta (Figure 4J). The specificity of the Cx43 antibody was verified by the detection of this connexin within the junction zones in rat ventricular myocytes (Figure 5K), which lack Cx40 and Cx37 [26].

### Vascular Connexin Expression in Response to Blood Flow Alteration

As outlined above, the venous graft was exposed to pronounced alterations in mechanical forces, which were even larger than in the femoral artery. Interestingly, these hemodynamic changes modified the expression of Cx at the transcriptional level. Two weeks after implantation of the graft, levels of Cx37 mRNA in the graft remained unchanged compared to the sham operated control group, whereas the levels of mRNA Cx40 decreased substantially (Figure 5). In contrast to Cx37 and Cx40, Cx43 mRNA expression was significantly increased (nearly 2-fold, Figure 5).

**Figure 6:**
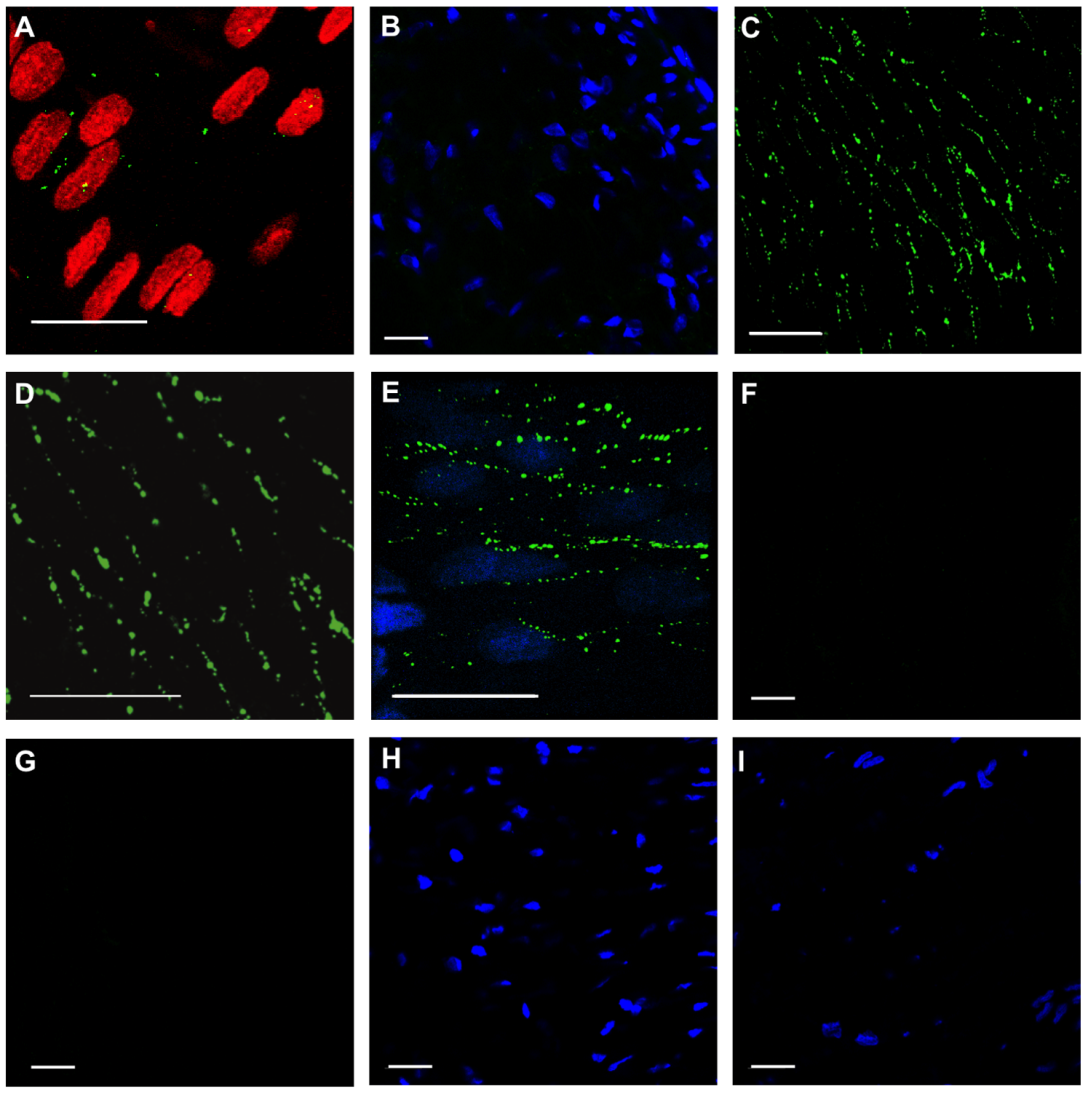
Femoral veins harvested 5 days after AV loop interposition being exposed to hemodynamic alteration exhibited strongly enhanced Cx43 expression (C–E) compared to control femoral veins exposed to their physiologic blood flow (A) and to veins that where dissected and reintegrated into the venous limb of the circulation by microsurgical techniques (B, sham operated group). Cx43 (green) was homogeneously distributed along endothelial cell borders of the venous graft (nuclei stained in blue in E). Cx37 (F) and Cx40 (G) were not detectable as in control veins. Negative controls revealed absence of non-specific staining by the secondary antibodies (H: Alexa Fluor 488, I: Alexa Fluor 594). Nuclei shown in red or blue, Cx in green, Scale bars: 20 µm.

The protein expression of Cx37 and Cx40, which was hardly detectable in untreated veins, was similarly weak in grafted veins. Alterations of these Cx isoforms were thus not detected by whole mount immunolabeling after 5 days of AV loop flow exposure (Figure 6F and 6G). In contrast, Cx43 staining was strongly enhanced in grafted veins after an exposure of 5 days i.e. at a timepoint before angiogenesis is observed [22]. Cx43 was located homogenously distributed in the plasma membrane along cell borders (Figure 6C, 6D and 6E), which are identified according to their shape and their nuclei as endothelial cells. Cx43 staining in these exposed veins resembled the staining pattern of Cx40 or Cx37 in untreated femoral arteries as well as the Cx43 staining pattern observed in the thoracic aorta as shown in Figure 4. In contrast, Cx43 was only rarely detected in untreated veins (Figure 6A) and sham operated control veins (Figure 6B).

## Discussion

Using an established model to modulate hemodynamic forces acting on a venous graft interpositioned to create an arteriovenous loop we demonstrate that the imposed substantial hemodynamic changes result in considerable modulation of connexin expression in the femoral vein. A striking finding is the strong upregulation of Cx43 expression that precedes the subsequently ensuing angiogenesis which was described in this model previously [22]. Most interestingly, Cx43 expression appears to be specifically enhanced in endothelial cells, while it is detected only marginally at physiologic conditions in this vein. Although the idea that Cx43 expression is increased in regions of high and disturbed flow in vivo has already been suggested [19] the arteriovenous graft used in the present manuscript provides a reliable model to show a direct correlation between Cx43 expression and high flow conditions. The present study emphasizes that mechanical loads modulate the regulation of Cx43 expression also in vivo in venous vessels subjected to arterial hemodynamics which was observed previously in vitro in cell culture [18]. Interestingly, Cx43 hemichannels in mechanosensory osteocytes also respond to mechanical stimulation leading to the opening of hemichannels which is mediated through a direct interaction of integrin alpha5beta1 with Cx43 [27].

We characterized herein the underlying hemodynamic forces acting on the venous graft, which was previously identified as the effector of the early AV loop neovascularisation [22]. In untouched femoral vessels mean blood flow was expectedly similar in artery and vein but flow was substantially more pulsatile in arteries. The interposition of a venous graft between artery and vein resulted in a loss of vascular resistance and introduced a 4.5-fold increase in blood flow through the grafted vessels. The intervention also exposed the venous graft to a drastically increased pulsatile flow that even exceeded arterial pulsatility. This verifies the substantial hemodynamic changes that are expected in a vessel serving as an AV loop.

After 15 days, a large network of new vessels was observed within the 3-dimensional construct by means of micro-CT which arose from the venous graft and was perfused as evidenced by staining following ink injection. During this period of neovascularization blood flow through the graft decreased and reattained the level of non-treated vessels. The substantial decrease of flow reflects a pronounced increase of resistance along the grafted vessel and was associated with a reduction of flow amplitude. Across the loop, the flow amplitude decreased and thus was significantly lower at its outlet suggesting that the blood perfused at least partially a microcirculatory bed, most likely the newly formed vessels.

The detailed evaluation of Cx expression in the grafted vessels revealed substantial changes at the transcriptional and protein level. In untreated vessels, Cx37 and Cx40 were found to be expressed in the endothelium of the femoral artery, whereas these Cx were only merely detectable in the femoral vein which is in line with previous studies [28]–[30]. Expression of Cx43 was weak in both femoral vessels, but it was, together with Cx40 and Cx37, expressed in the endothelium of the thoracic rat aorta reflecting a non-uniform expression pattern of Cx43 depending on vessel type and possibly function. Hong and Hill also reported distinct Cx43 expression along the rat arterial system and demonstrated that its expression in both vascular cell types (endothelium and smooth muscle) is restricted to elastic arteries but Cx43 is also expressed in specific muscular arteries (mesenteric, hepatic, tail) solely in endothelial cells [31]. This distinct Cx43 expression may be related to local flow pattern and pronounced at sites and in vessels exhibiting more oscillatory, disturbed flow, like large elastic arteries [19].

In veins subjected to enhanced flow and pulsatility, Cx37 mRNA remained unchanged and Cx40 mRNA decreased. Immunostaining did not reveal obvious modifications in protein levels of Cx37 and Cx40, which were rather weak in these grafted and also in control veins. In marked contrast, Cx43 expression was enhanced at the mRNA as well as the protein level in grafted vessels and Cx43 was localized in endothelial cell membranes. Since angiogenic factors were not added and ischemic tissue is not present in the current model, we suggest that the hemodynamic alteration is causal for the upregulation of Cx43 in endothelial cells in grafted veins and its integration into the membrane. Previously, it was suggested that pulsatility of flow is central for arterial identity and related to Cx40 expression [17]. Our findings support and extend this idea as the present study is the first to demonstrate that a vein exposed to large oscillations of flow adopts a phenotype of Cx43 expression that resembles that of a vessel subjected physiologically to such flow patterns (i.e. the thoracic aorta). The hypothesis that altered hemodynamics and possibly turbulent flow drive endothelial Cx43 expression is in line with observations of localized expression of Cx43 at sites of disturbed flow in the rat aortic endothelium [19]. In vitro studies have further suggested mechanical strain leading to cellular stretching may also alter Cx43 expression, preferentially in smooth muscle cells [18]. We cannot distinguish from our present data between turbulent flow or strain as the causal stimulus because the grafted vein was very likely also exposed to enhanced oscillatory strain. Currently, it remains unclear why Cx40 and Cx37 expression was unaltered as Cx40 was previously shown to be upregulated by flow [17]. It is tempting to speculate that laminar flow and shear stress is a prerequisite for the expression of these two Cx which are predominantly expressed in endothelial cells of arterial vessels throughout the circulation. Such conditions did not prevail in the current model at the time point Cx expression was evaluated according to our hemodynamic data.

The increase of Cx43 was observed already on day 5, a time point at which angiogenesis is still lacking [22]. Since the grafted vein is the source of angiogenesis [22] it is tempting to speculate that the upregulation of Cx43 is linked to this vessel growth because Cx43 was recently demonstrated to be required for angiogenesis in the embryo and uterus during pregnancy. The conditional deletion of Cx43 resulted in an impaired production of several key angiogenic factors, including VEGF in the embryo and uterus [8]. Additionally, down-regulation of Cx43 by means of small interference RNA decreased proliferation and angiogenesis in human aortic endothelial cells in vitro [9]. Together, this suggests a role for Cx43 in angiogenesis in the context of hemodynamic changes. This function may relate to communication through Cx43 gap junctions channels and subsequent altered neutrophil migration [32]. However, non-gap junctional effects, e.g. Cx43 serving as a hemichannel releasing mediators may also be involved. Recently, interactions of Cx with cytoskeletal proteins have been implicated in modulating cell migration and growth control, which would be even more intriguing and provide completely channel-independent regulatory pathways [33]–[36]. Based on the present data it is too speculative to suggest that the upregulation of Cx43 is involved in the angiogenesis. However, the grafted vein model is well accessible for elaborated imaging techniques and allows local application of various compounds to explore these interactions.

In summary, we present evidence that Cx43 expression is strongly upregulated in a grafted vein exposed to large oscillatory flow and demonstrate a phenotypic change of a vessel in vivo with respect to Cx expression. The subsequently ensueing angiogenesis that is observed independent of angiogenic factors may be a consequence of this phenotypic change. This observation may have implications in diseases in which angiogenesis is a therapeutical measure.
